# Genome Sequence of a *Clostridium neonatale* Strain Isolated in a Canadian Neonatal Intensive Care Unit

**DOI:** 10.1128/genomeA.01431-15

**Published:** 2016-01-21

**Authors:** Samia Benamar, Nadim Cassir, Bernard La Scola

**Affiliations:** Aix Marseille Universite, Institut Hospitalo-Universitaire Méditerranee Infection, Unité de Recherche sur les Maladies Infectieuses et Tropicales Emergentes (URMITE), UM63, CNRS, INSERM, IRD 198 7278 1095, Faculté de Médecine de Marseille, Marseille, France

## Abstract

*Clostridium neonatale* is a Gram-positive endospore-forming obligate anaerobe first isolated from the feces of premature neonates during an intensive care unit outbreak of necrotizing enterocolitis (NEC) in a Canadian neonatal intensive care unit. Here, we announce the genome draft sequence of this bacterium. It is composed of 4,710,818 bp and contains 4,169 protein-coding genes and 80 RNA genes, including 11 rRNA genes.

## GENOME ANNOUNCEMENT

*Clostridium neonatale* was first isolated from the blood and feces from neonates born between 30 weeks and term gestation in a context of a necrotizing enterocolitis epidemic affecting 8 neonates (1). *C. neonatale* was isolated from stool samples and rectal samples, which were inoculated onto prereduced blood agar supplemented with vitamin K and hemin plates and incubated at 37°C in an anaerobic chamber (1). The 16S rRNA sequence of this bacterium showed 98% identity using BLASTN (2) with the sequence of *Clostridium saccharobutylicum* strain NCP 262. The genus *Clostridium* has been associated with several diseases in adults and children, and the species *Clostridium butyricum* was specifically associated recently with cases of necrotizing enterocolitis (3). The presence of four toxins detected in the genomes of these *C. butyricum* strains was hypothesized to be responsible for the disease (3).

The genome of *C. neonatale* (ATCC BAA-265) was sequenced using MiSeq technology (Illumina, San Diego, CA, USA), with a mate-pair 250-bp strategy. Velvet version 1.1.06 (4) was used to perform a *de novo* assembly of the reads. The total estimated size of the *C. neonatale* genome is 4.71 Mb (coverage, 41×), composed of 20 contigs regrouped into 17 scaffolds, with an average G+C content of 28.42%. Using BLASTN and RNAmmer (5), the genome was shown to contain 80 RNA genes, including eight 5S rRNA genes, three 16S rRNA genes, and 69 tRNA genes.

The draft genome sequence of *C. neonatale* is smaller than those of *Clostridium beijerinckii* and *Clostridium diolis* (4.71, 6.00, and 5.85 Mb, respectively) but larger than those of *Clostridium paraputrificum*, *Clostridium sartagoforme*, and *Clostridium perfringens* (3.56, 3.98, and 3.26 MB, respectively). The G+C content of *C. neonatale* is smaller than those of *C. paraputrificum* and *C. beijerinckii* (29.64 and 29.86%, respectively) but larger than those of *C. sartagoforme* and *C. perfringens* (27.91 and 28.38%, respectively).

Potential coding sequences (CDSs) were predicted using the Prodigal software (6). Assignment of protein functions was performed by searching against the GenBank, Clusters of Orthologous Groups (COG), and Pfam (7) databases using BLASTP. A total of 4,249 genes were identified, representing a coding capacity of 3,907,356 bp (82.94% of the sequenced genome). Among these genes, 2,638 (63.27%) genes were associated with COG categories (8). The majority of the gene products are involved in the metabolism of amino acids and carbohydrates, ribosomal structure and biogenesis, and transcription and translation. We identified 1,780 proteins associated with a mobilome found in the phage database (PhAnToMe) and 119 toxin/antitoxin protein predictions. Interestingly, the four toxins described in *C. butyricum* and that are suspected to be the cause of necrotizing enterocolitis (3) were also identified in *C. neonatale* (identity percent ranging from 77% to 92%).

### Nucleotide sequence accession numbers. 

The *C. neonatale* genome was deposited in EMBL under the accession numbers CZLD01000001 to CZLD01000020.
